# Resveratrol, MicroRNAs, Inflammation, and Cancer

**DOI:** 10.4061/2011/102431

**Published:** 2011-08-10

**Authors:** Esmerina Tili, Jean-Jacques Michaille

**Affiliations:** ^1^Department of Molecular Virology, Immunology, and Medical Genetics, Ohio State University, Biomedical Research Tower, 460 W 12th Avenue, Columbus, OH 43210, USA; ^2^LBMN-INSERM U866, Université de Bourgogne, Faculté Gabriel, 6 boulevard Gabriel, 21000 Dijon, France

## Abstract

MicroRNAs are short noncoding RNAs that regulate the expression of many target genes posttranscriptionally and are thus implicated in a wide array of cellular and developmental processes. The expression of *miR-155* or *miR-21* is upregulated during the course of the inflammatory response, but these microRNAs are also considered oncogenes due to their upregulation of expression in several types of tumors. Furthermore, it is now well established that inflammation is associated with the induction or the aggravation of nearly 25% of cancers. Therefore, the above microRNAs are thought to link inflammation and cancer. Recently, resveratrol (trans-3,4′,5-trihydroxystilbene), a natural polyphenol with antioxidant, anti-inflammatory, and anticancer properties, currently at the stage of preclinical studies for human cancer prevention, has been shown to induce the expression of *miR-663*, a tumor-suppressor and anti-inflammatory microRNA, while downregulating *miR-155* and *miR-21*. In this paper we will discuss how the use of resveratrol in therapeutics may benefit from the preanalyses on the status of expression of *miR-155* or *miR-21* as well as of *TGF**β**1*. In addition, we will discuss how resveratrol activity might possibly be enhanced by simultaneously manipulating the levels of its key target microRNAs, such as *miR-663*.

## 1. Inflammation and Cancer

Inflammation represents a complex, nonspecific immune response of the body to pathogens, damaged cells, tissue injury, allergens, toxic compounds, or irritant molecules [1]. While it is normally self-contained, it may become permanent and chronic. It may also escape the original tissue and spread via the circulatory and/or the lymphatic system to other parts of the body, producing a systemic inflammatory response syndrome such as sepsis in case of an infection. Chronic inflammation is associated with a simultaneous destruction and healing of the tissue from the inflammatory process and has been linked to a number of pathologies, including cancer, chronic asthma, rheumatoid arthritis, multiple sclerosis, inflammatory bowel diseases, and psoriasis, as well as several types of neurological disorders. The inflammatory response is coordinated by a large range of mediators that form complex regulatory networks [1]. The recruitment of leukocytes in the peripheral tissues is the hallmark of inflammation. It is mediated by several types of chemokines [1–3], which act through their receptors located at the surface of the cytoplasmic membrane of leukocytes. The production of chemokines is induced by inflammatory stimuli such as bacterial lipopolysaccharide (LPS), interleukin (IL)-1, or tumor necrosis factor (TNF). In addition, these chemokines have a clear role in angiogenesis and wound repair [4]. Finely tuned molecular mechanisms exist that ensure that the immune response required for the defense of the body has a limited duration and does not pass a certain maximum activation level, thus avoiding that, it becomes harmful to the organism. It is well acknowledged that unresolved immune response results in inflammation. 

Epidemiological studies suggest that as many as 25% of all cancers may be due to chronic inflammation [5–7]. The connection between inflammation and cancer consists of an extrinsic pathway, driven by inflammatory conditions that increase cancer risk, and an intrinsic pathway, driven by genetic alterations that cause inflammation and neoplasia [6]. Inflammatory mediators released by cancer-related inflammation induce genetic instability, leading to the accumulation of random genetic alterations in cancer cells [7]. The activation of Toll-like receptors (TLRs), a group of pattern recognition receptors functioning as sensors of pathogens and tissue damages, leads to the nuclear translocation of NF-*κ*B and the production of cytokines such as TNF, IL-1*α*, IL-1*β*, IL-6, and IL-8. However, TLR activation has been shown to accelerate the growth of adoptively transferred tumors [8–11]. Accordingly, the stimulation of TLRs leads to increased survival and proliferation of several cell lines [12, 13], and the intratumoral injection of *Listeria monocytogenes* induces TLR2 signaling in tumor cells, thus promoting their growth [14]. TLR signaling also enhances tumor cell invasion and metastasis by regulating metalloproteinases and integrins [15]. Chemokines also affect several tumor progression pathways, such as leukocyte recruitment and function, cellular proliferation, survival, or senescence, as well as invasion and metastasis and are the targets of a number of anticancer agents [5].

Tumor microenvironment contains various inflammatory cell types infiltrating the tumor area in response to inflammatory stimuli, such as macrophages, neutrophils, and mast cells [16, 17]. Tumor-associated macrophages (TAMs) are thought to play key roles in the production of various growth factors, angiogenic factors, proteinases, chemokines, and cytokines, through crosstalks with cancer cells and other tumor stromal cells [18–20]. Factors secreted by TAMs stimulate cell migration/motility, proliferation, survival, angiogenesis, and metastasis, resulting in a dynamic environment that favors the progression of cancer, thus affecting the clinical outcome of malignant tumors. TAMs have thus been described as “obligate partners for tumor-cell migration, invasion and metastasis” [21, 22]. Namely, in a genetic model of breast cancer in macrophage-deficient mice, the tumors developed normally but were unable to form pulmonary metastases in the absence of macrophages [23]. As tumor metastasis is responsible for approximately 90% of all cancer-related deaths, a better understanding of inflammation regulatory mechanisms may potentially allow to optimize the use of anticancer drugs that lower tumor-specific inflammatory response [18].

Finally, the transforming growth factor *β* (TGF*β*) regulates the immune response as well as the effects of the immune system on tumor progression or regression *in vivo* [24]. TGF*β* has been shown to suppress the antitumor activity of T cells, natural killer (NK) cells, neutrophils, monocytes, and macrophages, which together are able to promote or repress tumor progression depending on the cellular context [25–27]. Importantly, TGF*β*1, the most abundant and ubiquitously expressed isoform of TGF*β*, is usually considered a tumor-suppressor, due to its cytostatic activity in epithelia. However, on advanced stages of tumors, TGF*β*1 behaves as a tumor promoter, due to its capability to enhance angiogenesis, epithelial-to-mesenchymal transition, cell motility, and metastasis [28–30].

## 2. MicroRNAs and Inflammation

MicroRNAs (miRNAs) are short noncoding RNAs which regulate the translation and/or degradation of target messenger RNAs [31–33]. They have been implicated in the regulation of a number of fundamental processes, including muscle, cardiac, neural, and lymphocyte development, or the regulation of both the innate and adaptative immune responses [34, 35]. miRNAs originate from primary transcripts (pri-miRNAs) converted in the nucleus into precursor miRNAs (pre-miRNAs) by the RNase III Drosha, associated with DGCR8 to form the small microprocessor complex [36]. Pre-miRNAs are then exported in the cytoplasm where the miRNA hairpin is cleaved by the RNase III Dicer within the RISC loading complex. The guide strand, which corresponds to the mature miRNA, is then incorporated into the RISC complex [36]. miRNAs and their transcriptional regulators usually form autoregulatory loops aimed at controlling their respective levels [37]. miRNAs participate in many gene regulatory networks whose molecular malfunctions are associated with major pathologies such as cancer [34] or auto-mmune diseases [38–40].

Several miRNAs have been implicated in both inflammation and cancer [38–41]. The most prominent are* miR-155*, *miR-21,* and *miR-125b*. Thus the expression of *miR-155* is strongly elevated in several human leukemias and lymphomas ([40] and references therein). Transgenic mice with B cells overexpressing *miR-155* develop B-cell leukemia, and a sustained expression of *miR-155* in hematopoietic stem cells causes a myeloproliferative disorder [40]. On the other side, *miR-155* has been implicated in the regulation of myelopoiesis and erythropoiesis, Th1 differentiation, B-cell maturation, IgG1 production, somatic hypermutations, gene conversion, class switch recombination, and B- and T-cell homeostasis as well as in the regulation of the innate immune response [40]. Thus, *miR-155* levels increase following LPS treatment of Raw-264 macrophages, and *miR-155* transgenic mice show enhanced sensibility to LPS-induced endotoxin shock [42]. In contrast, *miR-155*-knock-out mice are unable to mount a proper T-cell or B-cell immune response [40]. The expression of another miRNA, *miR-125b*, was repressed within 1 hour of LPS challenge in Raw-264 cells [42]. In topic eczema, *miR-125b* expression was reduced in regions of the skin that were inflamed, while that of *miR-21* was enhanced [43]. Similarly, *miR-21* expression was increased by inflammation due to ulcerative colitis [44] and also by IL-13 and by specific antigens in OVA- and *Aspergillus fumigatus* antigens-induced asthma models [45]. The expression of *miR-21* changes dynamically during antigen-induced T-cell differentiation, with the highest levels of expression in the effector T cell [46]. *MiR-21* induction upon T-cell receptor (TCR) stimulation is believed to be involved in a negative feedback loop regulating TCR signaling [46]. *MiR-663* has drown recent attention due to its role not only as an anti-inflammatory miRNA but also as a tumor suppressor miRNA. Thus, *MiR-663* impairs the upregulation of *miR-155* by inflammatory stimuli [47]. In addition, the expression of this microRNA is lost in certain cancers such as gastric or pancreatic cancer, and it induces mitotic catastrophe growth arrest when its expression is restored in these cells [48]. 

## 3. MicroRNAs as Oncogenes and Tumor-Suppressor Genes

miRNAs participate in many gene regulatory networks whose molecular malfunctions are associated with cancers [35, 41]. Depending on the effects of their downregulation or overexpression, miRNAs have been described either as oncogenic (onco-miRs) or tumor suppressors. Thus, the *miR-17-92* cluster on chromosome 13, which contains six miRNAs (*miR-17*, *-18a*, *-19a*, *-20a*, *-19b-1,* and *-92a-1*), is amplified and overexpressed in B-cell lymphomas and solid tumors such as breast or small-cell lung cancers, where it may enhance oncogenesis by potentially targeting E2F1, p21/CDKN1A, and BCL2L11/BIM [49]. On the other hand, loss of function of *miR-17-92* miRNAs might be advantageous for cancer cells in certain settings. Namely, loss of heterozygosity at the 13q31.3 locus has been observed in multiple tumor types, and a genome-wide analysis of copy number alterations in cancer revealed that the *miR-17-92* cluster was deleted in 16.5% of ovarian cancers, 21.9% of breast cancers, and 20% of melanomas [50]. In contrast, the twelve members of the human *let-7* gene family are frequently downregulated in cancers like lung, colon, or other solid tumors [34, 35]s and are, therefore,s considered as tumor-suppressor miRNAs in these types of cancers. In particular, *let-7* miRNAs target oncogenes of the *Ras* family [51] and c-Myc, and their expression in colon tumors results in reduced levels of both Ras and c-Myc [52]. *miR-21* is overexpressed in several cancers, including colorectal carcinomas (CRCs), gliomas, as well as breast, gastric, prostate, pancreas, lung, thyroid, and cervical cancers [53, 54]. *miR-21* has been shown to function as an onco-miR, due to its targeting of transcripts encoding key regulators of cell proliferation and apoptosis such as PTEN and PDCD4 [53]. Beside *miR-21*, several miRNAs are overexpressed in CRCs, including *miR-17*, *miR-25*, *miR-26a,* and *miR-181a* [54, 55]. 

In human, the levels of both *miR-155* and *BIC* transcripts (that is, *miR-155* primary RNAs) are elevated in diffuse large B-cell lymphoma (DLBCL), Hodgkins lymphoma, and primary mediastinal B-cell lymphoma [40]. In contrast, a very weak expression of *miR-155* is found in most non-Hodgkins lymphoma subtypes, including Burkitt lymphoma. In addition, high levels of *BIC* and *miR-155* expression were reported in B-cell chronic lymphocytic leukemia (B-CLL) and in B-CLL proliferation centers [56]. Furthermore, *miR-155* was also upregulated in a subset of patients with acute myelomonocytic leukemia and acute monocytic leukemia. Accordingly, transgenic mice whose B cells overexpress *miR-155* developed polyclonal preleukemic pre-B-cell proliferation followed by B-cell malignancy [40]. On the other hand, it was reported that *BIC* cooperates with *c-Myc* in avian lymphomagenesis and erythroleukemogenesis [57]. Beside liquid malignancies, high levels of *miR-155* expression were found in solid tumors such as breast, colon, and lung cancers [40]. *miR-155* was recently shown to induce a mutator phenotype by targeting Wee-1, a kinase regulating G_2_/M phase transition during the cell cycle [58]. Furthermore, it was shown that *miR-155* increases genomic instability by targeting transcripts encoding components of the DNA mismatch repair machinery [59]. As a consequence, the simultaneous *miR-155*-steered suppression of a number of tumor suppressor genes combined with a mutator phenotype might allow the shortening of steps required for tumorogenesis, and might also explain how chronic inflammation associated with high levels of *miR-155* induces cancer.

Finally, miRNAs have been implicated in metastasis. For example, it has been established that the downregulation of both *miR-103-1* and *miR-103-2* miRNAs induces epithelial-to-mesenchymal transition by targeting *Dicer1* transcripts [60]. Furthermore, several miRNAs, including *miR-21*, have been shown to activate metastasis by acting on multiple signaling pathways and targeting various proteins that are involved in this process. Thus, in breast cancer, which represents the most common malignancy among women in the world, miRNAs such as *miR-9*, *miR-10b*, *miR-21*, *miR-103/107*, *miR-132*, *miR-373,* and *miR-520* stimulate metastasis, while *miR-7*, *miR-30*, *miR-31*, *miR-126*, *miR-145*, *miR-146*, *miR-200*, *miR-205*, *miR-335*, *miR-661,* and miRNAs of the *let-7* families in contrast impair the different steps of metastatic process, from epithelial-to-mesenchymal transition to local invasion to colonisation and angiogenesis [61].

Numerous reports have provided strong evidence that all of the above miRNAs potentially target a myriad of transcripts including those encoding transcription factors, cytokines, enzymes and kinases, implicated in both cancer and inflammation.

## 4. Anti-Inflammatory and Antitumor Properties of Resveratrol

Resveratrol (trans-3,4′,5-trihydroxystilbene) is a natural polyphenolic, nonflavonoid antioxidant found in grapes and other berries, produced by plants in response to infection by the pathogen *Botrytis cinerea* [62]. Recent studies have documented that resveratrol has various health benefits, such as cardiovascular- and cancer-preventive properties [63–65], and this compound is currently at the stage of preclinical studies for human cancer prevention [66, 67]. Resveratrol was first shown to inhibit both tumor promotion and tumor progression in a mouse skin cancer model [68]. Resveratrol is also tested for preventing and/or treating obesity and diabetes [69, 70]. Fortunately, resveratrol toxicity is minimal, and even proliferating tissues such as bone marrow or intestinal tract are not adversely affected [71].

Resveratrol exerts its effects at multiple levels. Both its *m*-hydroxyquinone and 4-hydroxystyryl moieties have been shown to be important for the determination of resveratrol inhibitory properties toward various enzymes. This include lipoxygenases and cyclooxygenases that synthesize proinflammatory mediators from arachidonic acid, protein kinases such as PKCs and PKD, and receptor tyrosine kinases, lipid kinases, as well as IKK*α*, an activator of NF-*κ*B pathway, which establishes a strong link between inflammation and tumorigenesis [72]. Also, resveratrol inhibition of P450/CYP19A1/Aromatase, by limiting the amount of available estrogens and consequently the activity of estrogen receptors, has been proposed to contribute to the protection against several types of cancer, including breast cancer [72, 73]. Of note, resveratrol also inhibits the formation of estrogen-DNA adducts, which are elevated in women at high risk for breast cancer [74].

Resveratrol in addition regulates apoptosis and cell proliferation. It induces growth arrest followed by apoptotic cell death and interferes with cell survival by upregulating the expression of proapoptotic genes while simultaneously downregulating the expression of antiapoptotic genes [75]. Resveratrol induces the redistribution of CD95 and other death receptors in lipid rafts, thus contributing to their sensitization to death receptor agonists [75]. It also causes growth arrest at G1 and G1/S phases of cell cycle by inducing the expression of CDK inhibitors p21/CDKN1A and p27/CDKN1B [63]. In addition, resveratrol directly inhibits DNA synthesis by diminishing ribonucleotide reductase and DNA polymerase [72, 76, 77]. Altogether, antiproliferative activities of resveratrol involve the differential regulation of multiple cell-cycle targets in a cell-type-dependent manner [72, 75].

One of the possible mechanisms for resveratrol protective activities is by downregulation of the inflammatory responses [78]. That includes the inhibition of synthesis and release of proinflammatory mediators, modifications of eicosanoid synthesis, or inhibiting the enzymes, such as cyclooxygenase-1 (COX-1/PTGS1) or -2 (COX-2/PTGS2), which are responsible for the synthesis of proinflammatory mediators, through the inhibitory effect of resveratrol on transcription factors like NF-*κ*B or activator protein-1 (AP-1) [78, 79]. Of note, constitutive COX-2 expression generally predicts aggressiveness of tumors, therefore, the use of nonsteroidal anti-inflammatory drugs that inhibit COX-2 in cancer treatment. However, cytoplasmic COX-2 can relocalize in the nucleus. This nuclear relocalization of COX-2 is induced by resveratrol, and exposure of resveratrol-treated cells to a specific COX-2 inhibitor blocked resveratrol-induced apoptosis, indicating that COX-2 displays proapoptotic activity in the nucleus, which may be associated with the generation of complexes of COX-2 and ERK1/2 mitogen-activated protein kinases. In mouse macrophages, resveratrol also displays antioxidant activity, decreasing the production of reactive oxygen species and reactive nitrogen species and inhibiting nitric oxygen synthetase (NOS)-2 and COX-2 synthesis as well as prostaglandin E2 production [80].

Furthermore, in 3T3-L1 adipocytes, resveratrol inhibits the production of the TNF-induced monocyte chemoattractant protein (MCP)-1/CCL2. MCP-1 plays an essential role in the early events during macrophage infiltration into adipose-tissue, which results in chronic low-grade inflammation, a key feature of obesity type 2 diabetes characterized by adipose tissue macrophage infiltration and abnormal cytokine production [81]. Finally, it is also well established that some anti-inflammatory effects of resveratrol arise from its capability to upregulate histone deacetylase sirtuin 1 (SIRT1) activity, that also presents antitumor and anti-inflammatory capabilities [82]. Altogether, it is clear that the key antitumor properties of resveratrol [77–82] are linked to its anti-inflammatory effects.

The fact that resveratrol targets, directly or indirectly, so many different factors, exerts such a wide influence on cell homeostasis, and provides such a range of health benefits, suggested that some of its effects should arise from its capability to modulate the activity of global regulators. Furthermore, the ability of each miRNA to potentially regulate the levels and, therefore, the activity, of tens to hundreds of target genes, strongly suggested that resveratrol should be able to modify the composition of miRNA populations. Indeed, it has recently been shown that resveratrol decreases the levels of several proinflammatory and/or oncogenic miRNAs and upregulates miRNAs with anti-inflammatory and/or antitumor potentials [47, 83].

## 5. *MiR-663* as a Mediator of Resveratrol Anti-Inflammatory Activity

Affymetrix microarrays and RNase-protection assays showed that resveratrol treatment of human THP-1 monocytic cells upregulated the expression of *LOC284801* transcripts, that contain the sequence of *pre-miR-663* and thus represent *miR-663* primary transcripts. MiRNA microarrays and RNase-protection assays accordingly confirmed these data [47]. Interestingly, *in silico* analysis using TargetScan (http://www.targetscan.org/) suggested that *miR-663* may potentially target transcripts encoding factors implicated in (i) the mounting of the immune response, especially JunB, JunD, and FosB, which encode AP-1 factors known to activate many cytokine genes in partnership with NFAT factors [84], (ii) TLR signaling, such as the kinases RIPK1 and IRAK2, and (iii) the differentiation of monocytes, Th1 lymphocytes, and granulocytes.

An antisense *miR-663* inhibitory RNA (*663-I*) proved capable of increasing global AP-1 activity in unchallenged THP-1 cells, showing that *miR-663* indeed target transcripts encoding AP-1 factors in these cells. These effects were in particular directed toward *JunB* and *JunD* transcripts [47]. In agreement with previous results [79], resveratrol blocked the surge of AP-1 activity that occurs following LPS challenge due to the fact that *JunB* transcripts peak within the first hour, leading to the accumulation of JunB in the next few hours [85]. This inhibitory effect of resveratrol on AP-1 activity was partly impaired by *663-I*, indicating that it arises at least in part from the upregulation of *miR-663* by resveratrol [47]. Western blots showed that resveratrol impaired JunB neosynthesis, while still allowing the phosphorylation, that is, the activation of JunB following LPS treatment to take place, at least to a certain extent. Given that AP-1 factors include c-Jun, JunB, JunD, FosB, Fra-1, and Fra-2, as well as Jun dimerization partners JDP1 and JDP2 or the closely related ATF2, LRF1/ATF3, and B-ATF, so that potentially about 18 different dimeric combinations may be formed, the capability of resveratrol to specifically target a subset of AP-1 dimers through the upregulation of *miR-663* might have profound effects on the levels of the transcriptional activity of promoters to whom different AP-1 factors can compete for binding. Due to the many roles of AP-1 factors both in inflammation and cancer [86, 87], the specific targeting of genes encoding a subset of AP-1 factors, by changing the composition of AP-1 dimers on key promoters, may possibly explain some of the multiple anti-inflammatory and anticancer properties of resveratrol.

Of note, *miR-155* had been shown to be under AP-1 activity in activated B cells [88]. Accordingly, *miR-663* reduced the upregulation of *miR-155* by LPS [42], which may be due to *miR-663* targeting of transcripts encoding JunB and JunD and also possibly FosB and KSRP, an RNA binding protein implicated in the LPS-induced *miR-155* maturation from its primary transcripts *BIC* [89]. This is of primary importance, for *miR-155* upregulation is the hallmark of inflammatory response following LPS treatment of macrophages/monocytes [42]. Resveratrol also dramatically impaired the upregulation of *miR-155* by LPS, an effect partly inhibited by *663-I* [47]. Altogether, these results indicate that the anti-inflammatory properties of resveratrol arise, at least in part, from its upregulation of *miR-663* and its downregulating effects on *miR-155* and that *miR-663* might possibly qualify as an anti-inflammatory miRNA.

## 6. MicroRNAs as Mediators of Resveratrol Anticancer Effects

The results reported here above also suggested that, due to its targeting of AP-1 factors, known to play a role in tumorigenesis and cell invasion [86, 87] and due to its downregulation of *miR-155*, whose levels increase in solid as well as in liquid tumors [40], *miR-663* may also possibly provide resveratrol with some of its anticancer properties. Namely, *miR-663* was found to be downregulated in hormone refractory prostate cancer cells, along with *miR-146a* and *miR-146b *[90], further supporting the hypothesis that this miRNA is a tumor-suppressor gene whose one of the function is to keep low the expression level of oncogenic *miR-155* [47, 48].

CRC is the third most common malignancy and the fourth biggest cause of cancer mortality worldwide [91, 92]. Despite the increased use of screening strategies such as fecal occult blood testing, sigmoidoscopy, and colonoscopy, more than one-third of patients with colorectal cancer will ultimately develop metastatic disease [92]. On the other hand, the TGF*β* signaling pathway is one of the most commonly altered cellular signaling pathways in human cancers [93]. Among the three TGF*β* isoforms expressed in mammalian epithelia (TGF*β*1, TGF*β*2, and TGF*β*3), TGF*β*1 is the most abundant and ubiquitously expressed one. TGF*β* signaling is initiated by the binding of TGF*β* ligands to type II receptors (TGF*β*R2). Once bound by TGF*β*, TGF*β*R2 recruits, phosphorylates, and thus activates the type I TGF*β* receptor (TGF*β*R1). TGF*β*R1 then phosphorylates two transcriptional regulators, namely, SMAD2 and SMAD3, which subsequently bind to SMAD4. This results in the nuclear translocation of SMAD complexes, allowing SMADs to interact with transcription factors controlling the expression of a multitude of TGF*β* responsive genes [94]. The expression of TGF*β*1 in both tumor and plasma was found to be significantly higher in patients with metastatic colorectal cancer, and increasing colorectal tumor stage was correlated with higher TGF*β*1 expression in tumor tissues [95].

miRNA microarrays recently showed that resveratrol treatment of SW480 human colon cancer cells significantly increased the levels of 22 miRNAs while decreasing those of 26 others [83]. Among the miRNAs downregulated by resveratrol, *miR-17*, *miR-21*, *miR-25*, *miR-92a-2*, *miR-103-1* and *miR-103-2* have been shown to behave as onco-miRNAs, at least in certain contexts. Thus, genomic amplification and overexpression of *miR-17-92 *miRNAs is found in B-cell lymphomas as well as in breast and lung cancers [34, 54]. *MiR-21* is overexpressed in several cancers, including CRCs, gliomas, as well as breast, gastric, prostate, pancreas, lung, thyroid, and cervical cancers [53–55]. *MiR-17*, *miR-25*, *miR-26-a,* and *miR-181a* are also overexpressed in CRCs [34, 54]. In addition, several miRNAs, including *miR-21*, have been shown to activate metastasis by acting on multiple signaling pathways and targeting various proteins that are key players in this process [53]. Furthermore, the lower metastatic propensity of SW480 cells as compared with SW620 human colon cancer cells, both derived from the primary tumor and a metastasis of the same patient, respectively [96], was associated with a lower level of expression of *miR-103-1* and *miR-103-2*, two miRNAs that induce epithelial-to-mesenchymal transition by targeting *Dicer1* transcripts [60].

*In silico* analysis using TargetScan showed that miRNAs downregulated by resveratrol in SW480 cells potentially target transcripts encoding known tumor suppressor factors, such as the two antiproliferation factors PDCD4 and PTEN, the components of the mismatch repair machinery MLH3, MSH2 and MSH3, DICER1, the RNase III producing mature miRNAs from their immediate precursors in the cytoplasm, and several effectors and regulators of the TGF*β* signaling pathway [83]. Indeed, resveratrol treatment of SW480 cells lead to a greater accumulation of TGF*β*R1, TGF*β*R2, PDCD4, PTEN, and E-CADHERIN (a component of adherens junctions implicated in the maintenance of epithelial phenotype) [83]. Of note, among miRNAs upregulated by resveratrol, *miR-663* was the only one to target *TGF*β*1* transcripts. Luciferase assays and Western blots showed that resveratrol downregulated *TGF*β*1* in both a *miR-663*-dependent and a *miR-663*-independent manner [83]. Resveratrol treatment also decreased the transcriptional activity of SMADs under *TGF*β*1* signaling, an effect seemingly independent of *miR-663* [83].

Interestingly, it has been recently shown that GAM/ZNF512B, a vertebrate-specific developmental regulator first described in chicken [97], impairs the upregulation of miRNAs of the *miR-17-92* cluster by TGF*β*1 and that TGF*β*1 in turn downregulates *GAM*, at least in part through the upregulation of *miR-17-92* miRNAs [98]. The facts that *GAM* transcripts contain three consensus target sites for *miR-663* and that GAM is sensible to resveratrol treatment (Tili et al., unpublished results) raises the question of the possible existence of a gene regulatory network that would allow *miR-663* to impair GAM repressing activity on TGF*β*1 signaling pathway when TGF*β*1 works as a tumor suppressor, that is, at the early stages of tumorigenesis but not any more when this pathway starts to favor tumorigenesis and metastasis, that is, on advanced stages of cancers.

It is important to emphasize that TGF*β*1 has been shown to enhance the maturation of oncogenic *miR-21* through the binding of SMAD3 to *miR-21* primary RNAs [99] and also to increase the expression of *miR-155*, which is under the control of TGF*β*/SMAD activity [100]. Thus, by targeting AP-1 factors as well as TGF*β*1 and possibly SMAD3, *miR-663* might inhibit two of the pathways that upregulate *miR-155 *expression.

 The TGF*β* signaling pathway present multiple levels of regulation: sequestration of ligands into inactive precursor forms, ligand traps, decoy receptors, and inhibitory SMADs, not to mention the existence of SMAD-independent pathways and their interactions with many other critical signaling pathways which also proved to play a role in cancer. It is thus not surprising that the short (307-nt long) 3′-UTR of *TGF*β*1* transcripts contains a potential consensus target site for 28 miRNAs only. Of note, *TGF*β*1* 3′-UTR contains two target sites for two of these miRNAs and only one target site for 25 of the others. Therefore, the fact that *miR-663* may potentially target 5 different sites in *TGF*β*1* 3′-UTR suggests that this miRNA could represent a critical *TGF*β*1* regulator which may possibly be called upon action in emergency situations such as those when cells begin to proliferate anarchically or when a stronger immune response is required. The multiplicity of *miR-663* targets sites in *TGF*β*1* 3′-UTR further suggests that the effects of this miRNA might be both dose- and context-dependent, so that resveratrol effects on TGF*β*1 signaling pathway might well be also context-dependent. Finally, it is probable that, depending on the cell context, resveratrol might either increase the level of TGF*β* signaling—by inhibiting miRNAs targeting its main effectors—when it is beneficial to the organism, that is, when it works to maintain the integrity of epithelia and impair cell proliferation, or in contrast decrease TGF*β*1 signaling—by decreasing its production through the upregulation of *miR-663*—when TGF*β*1 starts to favor epithelial-to-mesenchymal transition and metastasis. For example, the targeting of both *TGF*β*1* and *SMAD3* transcripts might possibly allow resveratrol to impair TGF*β*1-induced SMAD3-dependent promotion of cell motility and invasiveness in advanced stages of gastric cancer [101, 102] or when SMAD2 and SMAD3 phosphorylated at both linker and COOH-terminal regions transmit malignant TGF*β*1 signal in later stages of human CRC [103].

## 7. Conclusions

It is notable that striking phenotypes are often driven through small changes in the cellular concentration of key factors. For example, in the B-cell compartment, *miR-150* curtails the activity of c-Myb transcription factor in a dose-dependent fashion over a narrow range of miRNA and c-Myb concentrations [104]. Thus, even slight effects of resveratrol on a handful of key miRNAs might well prove to be critical to its anti-inflammatory, anticancer and antimetastatic properties. In addition, the fact that *miR-663*, *miR-21*, *miR-155,* and TGF*β*1 have all been implicated in the regulation of cell proliferation, tumor apparition and development, metastasis formation, and innate immunity, strongly suggests that the capability of resveratrol to behave at the same time as an antitumor, antimetastatic, antiproliferation and anti-inflammatory agent most probably arises from its effects on the expression of a small set of critical endogenous miRNAs having the abilities to impact the cell proteome globally.

Finally, miRNAs have the promise to become biomarkers for different stages of cancer, both for diagnosis and prognosis. Furthermore, the discovery that resveratrol can modulate the levels of miRNAs targeting proinflammatory and/or protumor factors opens the possibility to optimize resveratrol treatments by manipulating in parallel the levels of expression of a few critical miRNAs. For example, from the experiments reported here above, it starts to become clear that the use of resveratrol would be especially beneficiary in the type of cancers where the TGF*β* pathway is implicated. Of course, resveratrol use would have to be carefully correlated with the stages of cancers, knowing that TGF*β* can have two faces, that is, anti- and prometastatic.

As a last remark, it should be noted that while resveratrol antitumor potential has been linked with data primarily from human cell culture systems, evidence that resveratrol can inhibit carcinogenesis in several organ sites emerged from results of cancer prevention and therapy studies in laboratory animal models [68]. Given that *miR-663* was only found in primates, the reports by Tili et al. [47, 83] come as a warning that studies in animal may not always allow to predict accurately the molecular effects of resveratrol in human, especially when it comes to miRNAs.
